# A Disease Model of Muscle Necrosis Caused by *Aeromonas dhakensis* Infection in *Caenorhabditis elegans*

**DOI:** 10.3389/fmicb.2016.02058

**Published:** 2017-01-04

**Authors:** Po-Lin Chen, Yi-Wei Chen, Chun-Chun Ou, Tzer-Min Lee, Chi-Jung Wu, Wen-Chien Ko, Chang-Shi Chen

**Affiliations:** ^1^Institute of Clinical Medicine, College of Medicine, National Cheng Kung UniversityTainan, Taiwan; ^2^Department of Internal Medicine, National Cheng Kung University Hospital, College of Medicine, National Cheng Kung UniversityTainan, Taiwan; ^3^Department of Internal Medicine, College of Medicine, National Cheng Kung UniversityTainan, Taiwan; ^4^Institute of Basic Medical Sciences, College of Medicine, National Cheng Kung UniversityTainan, Taiwan; ^5^Institute of Oral Medicine, College of Medicine, National Cheng Kung UniversityTainan, Taiwan; ^6^National Institute of Infectious Diseases and Vaccinology, National Health Research InstitutesTainan, Taiwan; ^7^Department of Biochemistry and Molecular Biology, College of Medicine, National Cheng Kung UniversityTainan, Taiwan

**Keywords:** *Caenorhabditis elegans*, *Aeromonas dhakensis*, disease model, muscle necrosis, infection

## Abstract

A variety of bacterial infections cause muscle necrosis in humans. *Caenorhabditis elegans* has epidermis and bands of muscle that resemble soft-tissue structures in mammals and humans. Here, we developed a muscle necrosis model caused by *Aeromonas dhakensis* infection in *C. elegans*. Our data showed that *A. dhakensis* infected and killed *C. elegans* rapidly. Characteristic muscle damage in *C. elegans* induced by *A. dhakensis* was demonstrated *in vivo*. Relative expression levels of host necrosis-associated genes, *asp-3*, *asp-4*, and *crt-1* increased significantly after *A. dhakensis* infection. The RNAi sensitive NL2099 *rrf-3 (pk1426*) worms with knockdown of necrosis genes of *crt-1* and *asp-4* by RNAi showed prolonged survival after *A. dhakensis* infection. Specifically knockdown of *crt-1* and *asp-4* by RNAi in WM118 worms, which restricted RNAi only to the muscle cells, conferred significant resistance to *A. dhakensis* infection. In contrast, the severity of muscle damage and toxicity produced by the *A. dhakensis* hemolysin-deletion mutant is attenuated. In another example, shiga-like toxin-producing enterohemorrhagic *E. coli* (EHEC) known to elicit toxicity to *C. elegans* with concomitant enteropathogenicty, did not cause muscle necrosis as *A. dhakensis* did. Taken together, these results show that *Aeromonas* infection induces muscle necrosis and rapid death of infected *C. elegans*, which are similar to muscle necrosis in humans, and then validate the value of the *C. elegans* model with *A. dhakensis* infection in studying *Aeromonas* pathogenicity.

## Introduction

Muscle necrosis is a necrotizing form of severe soft-tissue infection. These infections usually result in morbidity and mortality in humans even with early surgical intervention and antibiotic treatment. Necrotizing muscle infection is usually caused by toxin-producing bacteria, such as *Streptococcus*, *Staphylococcus*, *Clostridium*, *Vibrio vulnificus*, and *Aeromonas*, and characterized clinically by catastrophic progression of disease with severe tissue destruction (Anaya and Dellinger, 2007; Kuo et al., 2007; Hakkarainen et al., 2014).

Of note, the incidence of *Aeromonas* soft-tissue infections has been increasingly reported (Hiransuthikul et al., 2005; Chao et al., 2012; Chen et al., 2014a). *Aeromonas* can also cause wound infection or sepsis in burn patients (Barillo et al., 1996; Skoll et al., 1998; Kienzle et al., 2000). Cases of *Aeromonas* burn wound infections are often a result of water or soil exposure after the burn (Kienzle et al., 2000). Necrotizing fasciitis, myonecrosis, or both (myofascial necrosis) caused by *Aeromonas* usually progresses rapidly and with a fatal outcome even with antibiotic therapy and surgical intervention (Furusu et al., 1997; Tsai et al., 2007; Papadakis et al., 2012). Here, we specifically studied the pathogenesis of *A. dhakensis* in an animal host. The reasons for choosing *A. dhakensis* were: (1) *A. dhakensis* can cause severe soft-tissue infections in animal models and humans (Chen et al., 2014a,b); (2) *A. dhakensis* carries a number of virulence genes responsible for developing infectious diseases and its virulence has been recognized as the most potent among clinically important *Aeromonas* species (Figueras et al., 2009; Morinaga et al., 2013; Chen et al., 2014b); (3) Increasing evidence shows that *A. dhakensis* is widely distributed in the environment and causes a variety of infections in humans (Figueras et al., 2009; Aravena-Roman et al., 2011; Morinaga et al., 2013; Chen et al., 2014a). These findings suggest the clinical significance of this specific type of bacterial infection.

Small mammals, such as rats or mice, have been studied as experimental models of soft-tissue infection (Hidalgo-Grass et al., 2004; Cavanaugh et al., 2009; Chatterjee et al., 2016). Recently, non-mammalian models, such as *Drosophila* and *Caenorhabditis elegans*, have been increasingly used to study the pathology of soft-tissue infection (Zugasti et al., 2014; Zhang et al., 2015; Chatterjee et al., 2016). Among these models, *C. elegans* is attractive because of its suitability for studying host innate immunity (Kim, 2008; Marsh and May, 2012; Ermolaeva and Schumacher, 2014), convenience for gene analysis and observation, and a short life span.

*Caenorhabditis elegans* and *Aeromonas* are likely to compete in their aquatic environment and, therefore, have respective strategies to combat each other. In addition, despite their simplicity, worms have defined soft-tissues, such as epidermis and muscle bands similar to those in mammals and humans. Therefore, *C. elegans* can be a feasible model to study the pathogenesis of muscle necrosis. Our previous studies illustrated the applicability of virulence findings about *Aeromonas* species in *C. elegans* to mammalian cells, mice, and humans (Chen et al., 2014a,b). Clinical *Aeromonas* isolates that were virulent in *C. elegans* were also lethal to mice. The histological findings of mice with *Aeromonas* muscular infection, such as fragmented muscle fibers, edema of myocytes, and infiltration of inflammatory cells, resembled necrotizing myositis in humans (Chen et al., 2014b). However, the detailed histopathological characterization of muscle tissues in *C. elegans* with *Aeromonas* infection remains understudied.

In the present study, we demonstrated that *C. elegans* is a good surrogate model to study the pathogenesis of muscle necrosis caused by bacteria in humans. We also used this infection model to study the detrimental consequences of muscle necrosis in *C. elegans* after *A. dhakensis* infection.

## Materials and Methods

### *C. elegans* and Bacteria Strains

A wild-type Bristol N2 strain, NL2099 *rrf-3*(*pk1426*), WM118 *rde-1(ne300); neIs9[myo-3p*::HA::RDE-1 + *rol-6(su1006)]*, and RW1596 *myo-3(st386); stEx30[myo-3p::GFP + rol-6(su1006)]* of *C. elegans* were provided by the Caenorhabiditis Genetics Center (CGC), which is supported by the National Institutes of Health, Office of Research Infrastructure Programs (P40 OD010440). The NL2099 strain with homozygous *rrf-3* gene deletion allele was increasingly sensitive to RNAi when compared to wild-type animals (Simmer et al., 2002). The WM118 strain was used for muscle-specific RNAi (Qadota et al., 2007). The RW1596 strain with GFP-labeled sacromeres was used for analysis of muscle damage caused by *A. dhakensis* (Herndon et al., 2002). The animals were maintained on nematode growth (NG) plates containing *Escherichia coli* strain OP50 as the normal food source (Brenner, 1974).

A specific AAK1 strain of *A. dhakensis* (previously known as *A. aquariorum*; Beaz-Hidalgo et al., 2013) used in this study was a clinical isolate obtained from a patient with septicemia and necrotizing fasciitis, and its whole-genome sequences were deposited at DDBJ/EMBL/GenBank under accession nos. BAFL01000001 to BAFL01000036 and AP012343 (Wu et al., 2012).

### Plate Assay of *C. elegans* Infected by *A. dhakensis* AAK1

The plate assay, as previously described (Chen et al., 2010), was conducted to measure the life span of animals infected by *A. dhakensis*. Briefly, eggs were prepared by treating a population of *C. elegans* with hypochlorite/NaOH solution and transferring the resulting eggs to NG agar plates covered with *E. coli* OP50, which was washed in phosphate-buffered saline, grown in LB for 18–24 h at 37°C, and standardized to an OD_600_ of 2.0 for tests. When these worms reached the young adult stage, 150 nematodes were transferred to fresh plates, which represented the first day of life span analysis. Animals were transferred to fresh plates of *E. coli* OP50 or *A. dhakensis* AAK1 and monitored daily for dead animals. Animals that did not respond to gentle prodding and displayed no pharyngeal pumping were scored as dead. Animals that escaped from the plate or died due to internal hatching or protrusion of the gonads through the vulva were censored. Censored animals were included in the statistical analysis until the day of the censoring event.

### Life Span Assay with Heat-Killed Bacteria

*Escherichia coli* OP50 and *A. dhakensis* AAK1 were incubated with LB broth at 37°C for 18–24 h. The next day, bacterial broth was centrifuged to increase bacterial concentration to achieve an OD_600_ of 20.0. The concentrated bacteria were incubated at 65°C for 30 min to be inactivated. For the assays with heat-killed bacteria, 60 μl of heat-killed *E. coli* OP50 or heat-killed *A. dhakensis* AAK1 were placed on nematode growth media (NGM) and the worms were observed as described above.

### Liquid-Toxic Assay of *C. elegans* Infected with *A. dhakensis* AAK1

Following washing in phosphate-buffered saline, bacteria grown in LB for 18–24 h at 37°C and standardized to an OD_600_ of 3.0 were prepared for tests. To obtain a synchronously growing population, eggs were prepared by treating a population of *C. elegans* with hypochlorite/NaOH solution and transferring the resulting eggs to NGM plates covered with *E. coli* OP50, as previously described (Chen et al., 2014b). The synchronized adult L4 worms on NGM plates were washed in M9 buffer. After centrifugation, the pellets of worms were re-expanded with S medium, and 5 μl of solution containing approximately 30–40 worms were placed in each lawn of 48-well plates with 5 μl fluorodeoxyuridine (Sigma–Aldrich, Saint Louis, MO, USA) to prevent reproduction. Finally, 190 μl of bacteria in LB solution were added to achieve a total 200 μl in each lawn. Assay plates were incubated at 25°C for 3–6 days. The percentages of animal death were calculated as the numbers of dead animals/total animals found each day under a dissecting microscope.

### Live *C. elegans* Images

Synchronized late L4 to young adult stage N2 worms were plated on NGM plates seeded with *A. dhakensis* AAK1 or control plates seeded with *E. coli* OP50 at 25°C for 3 days. Stereo dissecting microscopic images were obtained using an Olympus SZX16 stereo room microscope with an Olympus DP72 cooled color digital (CCD) camera. The body length of N2 animals co-cultivated with *A. dhakensis* AAK1 or *E. coli* OP50 was measured for 4 days and compared by two-way ANOVA test.

### Morphological and Physiological Analyses: Muscle Damage, Pumping Rate, Body Length, and Brood Size

Adult RW1596 animals infected with *A. dhakensis* and *E. coli* OP50 for 48 or 72 h were observed by differential interference contrast (DIC) imaging with Nomarski optics and epifluorescence imaging with corresponding filters using a Nikon Eclipse Ti inverted microscope system with a DP72 CCD camera.

The pumping rate of *C. elegans* was measured by counting the contractions of the terminal pharyngeal bulb of each *C. elegans* worm. Ten L4 stage worms were transferred to NGM with *E. coli* OP50, *A. dhakensis* AAK1, heat-killed *E. coli* OP50, or heat-killed *A. dhakensis* AAK1 at first. The pumping times for each live worm were measured everyday by counting pumps every 10 s.

The body length of ten L4 stage *C. elegans* worms was measured for each group every day. Images were obtained from an Olympus microscope which was connected to a digital video camera using 2.5 times magnification and captured by IC capture 2.1 software. Body length was measured from head to tail tip and analyzed by the Image-Pro Plus software. The brood size of total progeny of *C. elegans* was counted for 4 days after infection. Ten worms were counted for each group.

### Transmission Electron Microscopy (TEM) and Scanning Electron Microscopy (SEM)

The adult N2 worms infected with *A. dhakensis* AAK1 and *E. coli* OP50 for 48 and 72 h were fixed and embedded separately using the standard methods described in the chapter “WormMethods” of the *WormBook*^1^ (accessed March 17, 2015) for TEM and SEM study. The thin sections were collected in longitudinal aspects and observed on a Hitachi H7650 transmission electron microscope. The surface of animals was observed on a JEOL USA JSM-6390LV scanning electron microscope.

### Measurement of Expression of Necrosis-Associated Genes

Expression of necrosis marker genes, *asp-3, asp-4*, and *crt-1*, was measured by quantitative real-time PCR (q-PCR) as described previously with some modifications (Wong et al., 1998). Approximately 10,000 L1 stage N2 worms were cultured on NGM plates with *E. coli* OP50. During the L4 to young adult stage, worms were transferred to either *E. coli* OP50 or *A. dhakensis* AAK1. After 72 h, animals were collected for RNA extraction. An RNA sample (2.0 μg) for each experimental group was converted to cDNA via reverse transcription. All q-PCRs were carried out using FastStart Universal SYBR Green Master (Rox) according to the manufacturer’s specifications and analyzed on a StepOnePlus Real-Time PCR System. Expression data were collected as Ct values, where Ct is equal to the number of PCR cycles required to amplify a given gene from a cDNA population. Changes of the expression of *asp-3* (forward primer: CCA TCC AGA GAA TCA AGC TCG; reverse primer: GGA GTA ATC AGA AAG ACC CTC G), *asp-4* (forward primer: CAT TTT GGC TCA ACC GTA ACC; reverse primer: CCT TGT CCA TCT TGA ATT GCC), and *crt-1* (forward primer: TCC AAT ACA CCG TCA AGC AC; reverse primer: AAT CTC CCA AGT CAG CAT CAG) were initially measured as ΔCt values which subsequently normalized against a housekeeping gene: *nhr-23* (forward primer: GCC GAA GAT GAT GCC GAG AT; reverse primer: GTC GCA GTG TCA AGA ATC CC). The fold-change values of the AAK1 group compared to OP50 group were estimated by following equation: Fold change = 2^[-ΔCt(AAK1)]^/2^[-ΔCt(OP50)]^.

### RNA Interference Assay

RNA interference (RNAi) clones to *asp-3, asp-4*, and *crt-1* were obtained from the *C. elegans* RNAi library (Ashrafi et al., 2003). *E. coli* HT115 with L4440, an empty vector, was used as a control of RNAi. Synchronized NL2099, or WM 118 L1 larvae were cultured on plates seeding *E. coli* HT115 with L4440, *asp-3, asp-4*, or *crt-1* RNAi at 20°C until the L4 stage. In the WM 118 strain, RNAi knockdowns were restricted specifically in muscle (Qadota et al., 2007). These L4 stage animals were transferred to plates together with *E. coli* HT115 either carrying RNAi plasmids or the L4440 plasmid together with *A. dhakensis* AAK1 at a ratio of 1:1. The animals were then incubated at 20°C and survival was observed.

### Statistical Analysis

Statistical analysis was performed and plotted using GraphPad Prism 5.0 (GraphPad Software, La Jolla, CA, USA). The Mantel-Cox log-rank test was used to assess statistical significance of difference in survival. The two-way ANOVA test was used assess statistical significance of difference in body length, brood size, and pharyngeal pumping rate analyses. The proportions of muscle damage for *A. dhakensis* and the control groups were compared by Mann–Whitney *U* test.

## Results

### *Aeromonas dhakensis* Infects and Kills *Caenorhabditis elegans*

**FIGURE 1:**
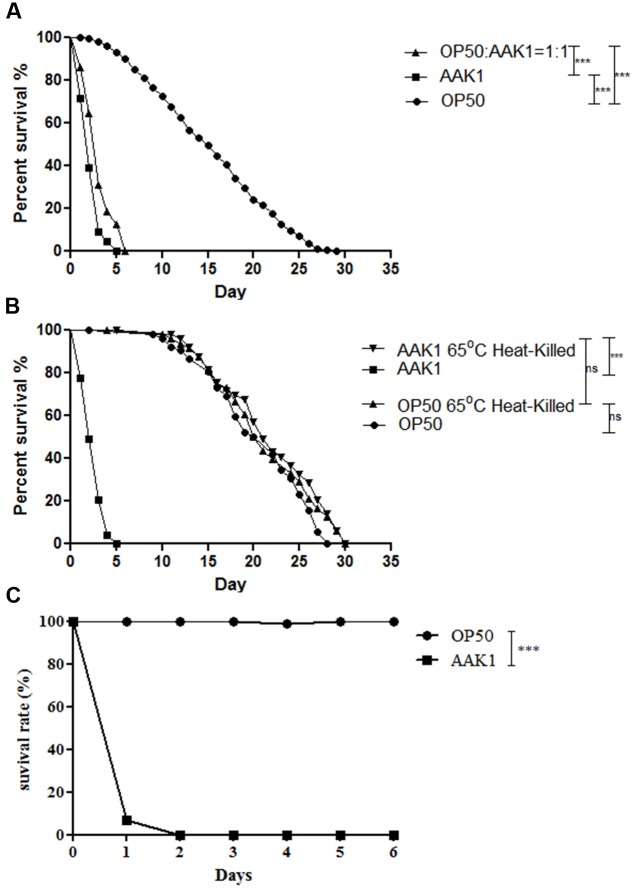
***Aeromonas dhakensis* shortens the life span of *Caenorhabditis elegans.*** **(A)** Worms infected with *A. dhakensis* and *A. dhakensis* mixed with *Escherichia coli* OP50 (1:1) had a significantly shorter life span when compared with control *E. coli* OP50. **(B)** *A. dhakensis* AAK1 showed toxicity when compared with heat-killed *A. dhakensis* or control *E. coli* OP50. **(C)** In liquid-toxic (LT) assay, the survival rate of *C. elegans* infected with *A. dhakensis* was significantly lower than those with control strain *E. coli* OP50. ^∗∗∗^*P* < 0.0001 by the Mantel-Cox log-rank test.

In the plate assay of *C. elegans*, clinical isolate *A. dhakensis* AAK1 significantly shortened the life span of *C. elegans* worms in comparison with the non-pathogenic control strain *E. coli* OP50, the normal laboratory food source of *C. elegans* (*P* < 0.0001; **Figure 1A**). *A. dhakensis* AAK1 mixed with *E. coli* OP50 in a ratio of 1:1 also decreased the life span of *C. elegans* worms when compared with *E. coli* OP50 (*P* < 0.0001). Moreover, heat-killed *A. dhakensis* AAK1 showed the toxicity to *C. elegans* similar to either OP50 or heat-killed bacteria (**Figure 1B**). Together these results suggested the short-lived phenotype of *C. elegans* worms feeding on *A. dhakensis* is not due to malnutrition but to the toxicity of *A. dhakensis.* In the liquid-toxic (LT) assay, the survival rate of *C. elegans* infected with *A. dhakensis* AAK1 was also significantly lower than those with *E. coli* OP50 (*P* < 0.0001, **Figure 1C**). Almost all animals were killed by *A. dhakensis* AAK1 at day 2 in the LT assay. Taken all together, our results show that *A. dhakensis* infects and kills *C. elegans.*

### Morphological Changes in *Caenorhabditis elegans* after *Aeromonas dhakensis* Infection

**FIGURE 2:**
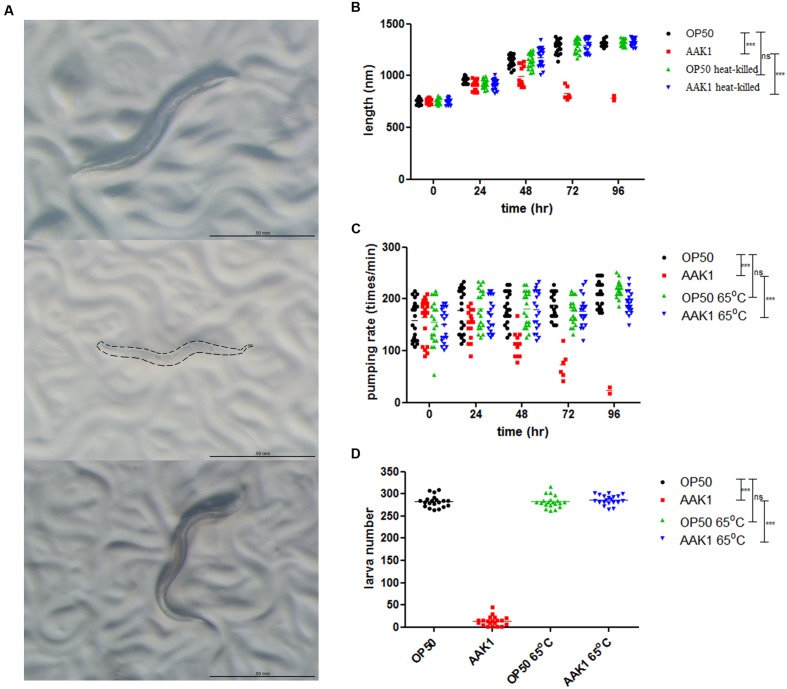
***Aeromonas dhakensis* infection induces morphological and physiological changes in *C. elegans*.** **(A)** Morphology of *C. elegans* treated with *A. dhakensis* AAK1 for 72 h (Top to bottom: *E. coli* OP50, *A. dhakensis* AAK1, 65°C heat-killed *A. dhakensis* AAK1). At 72 h, AAK1 infection resulted in total lysis of body. The corpse of AAK1 infected worm was marked by dashed line. The scale bar indicates 500 μm. **(B)** The body length of N2 *C. elegans* was significantly decreased after *A. dhakensis* AAK1 infection when compared with the control *E. coli* OP50 (*P* < 0.0001) by two-way ANOVA test. In contrast, 65°C heat-killed bacteria did not affect the size of the animals. The average feeding rate measured by pharyngeal movement (pumping times/min) **(C)** and brood size **(D)** of N2 worms infected with AAK1were significantly reduced, as compared with worms fed on OP50, OP50-65°C, or AAK1-65°C, ^∗∗∗^*P* < 0.0001.

Significant morphological changes in *C. elegans* were observed after infection with *A. dhakensis* AAK1 (**Figure 2**). At 72 h, *A. dhakensis* AAK1 caused total lysis of *C. elegans* (middle). In contrast, *C. elegans* morphology was normal, if *C. elegans* was fed with heat-killed *A. dhakensis* or *E. coli* OP50 (top and bottom). The average body length of N2 *C. elegans* significantly decreased after AAK1 infection when compared to worms fed on *E. coli* OP50, heat-killed *E. coli* OP50 (OP50-65°C), and heat-killed AAK1 (AAK1-65°C; *P* < 0.0001, at days 3 and 4; **Figure 2B**). The feeding rate of AAK1 infected worms obviously diminished. Pharyngeal muscle movement (pumping times/min) of N2 worms fed with OP50, AAK1, OP50-65°C, and AAK1-65°C were monitored. The average pumping rate of N2 *C. elegans* significantly reduced after AAK1 infection, as compared with worms fed on OP50, OP50-65°C, or AAK1-65°C (all *P* < 0.0001; **Figure 2C**). In addition, the reproduction of AAK1 infected worms significantly decreased. Total progeny numbers of N2 worms fed with OP50, AAK1, OP50-65°C, or AAK1-65°C were counted. The average brood size of N2 *C. elegans* was significantly reduced after AAK1 infection when compared with worms fed on OP50, OP50-65°C, or AAK1-65°C (all *P* < 0.0001; **Figure 2D**). These findings suggest that the morphology and behavioral changes of *C. elegans* were caused by *A. dhakensis* AAK1 infection.

**FIGURE 3:**
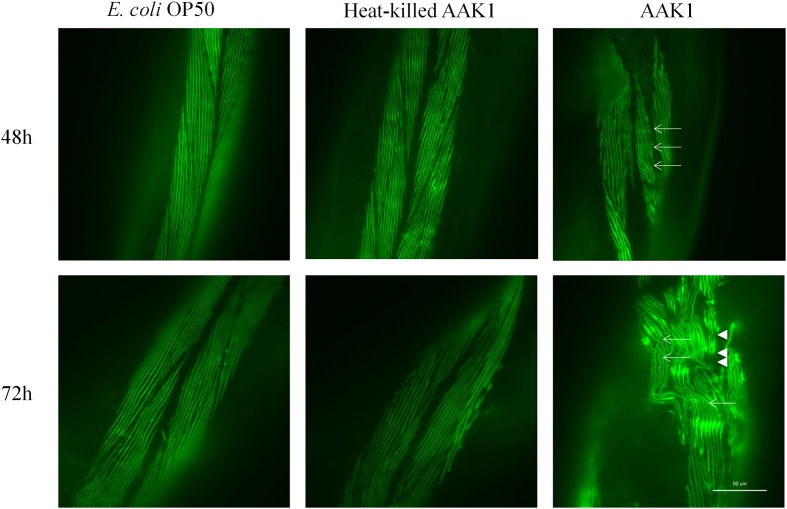
***Aeromonas dhakensis* induces muscle damage in *Caenorhabditis elegans*.** Bending of muscle fiber (arrows) was observed in worms with *A. dhakensis* AAK1 infection at 48 h. Rupture of muscle fibers (arrowheads) was obvious at 72 h. In contrast, *E. coli* OP50 or heat-killed *A. dhakensis* AAK1 did not cause muscle damage. Scale bar indicates 50 μm.

Muscle damage was observed in the transgenic *C. elegans* strain RW1596, in which sacromeres were labeled with MYO-3::GFP protein. Typical features of muscle damage, i.e., including bending and rupture of muscle fibers were observed at 48 and 72 h after *A. dhakensis* infection (**Figure 3**). The muscle damage caused by *A. dhakensis* in *C. elegans* became severe with time, manifesting obvious bending and rupture of muscles fiber at 72 h. In contrast, muscle fibers of *C. elegans* were intact either in the presence of *E. coli* OP50 or heat-killed *A. dhakensis* AAK1.

**FIGURE 4:**
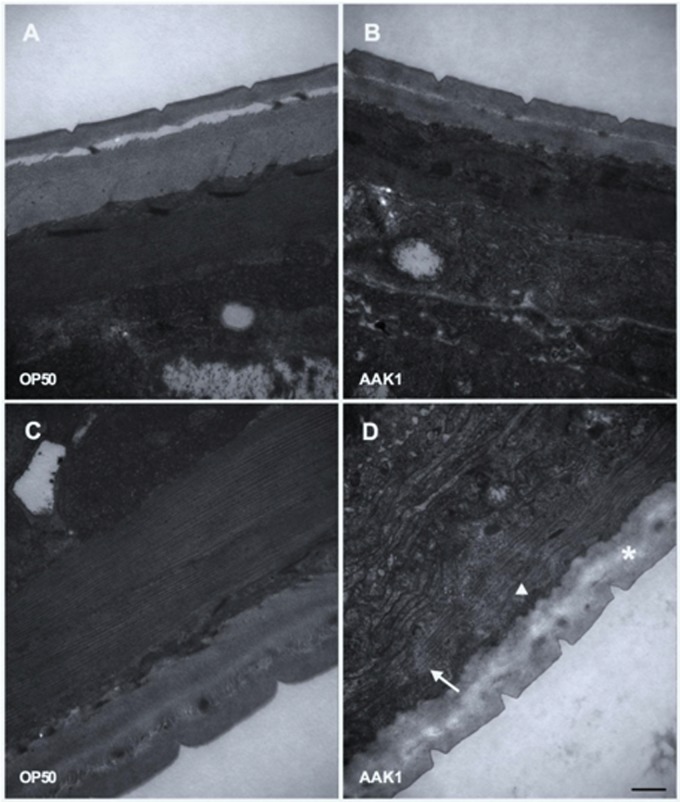
**Transmission electron microscopic analysis.** The morphological changes in *C. elegans* after feeding with *A. dhakensis* AAK1 (right panels) or *E. coli* OP50 (left panels) for 48 h (upper panels) or 72 h (lower panels) were observed with transmission electron microscopy **(A–D)**. Patch hypo-dense lesions (arrow), wavy change of myosin filaments (arrowhead), and decreased density of hypodermis (asterisks) were observed at 72 h after AAK1 infection **(D)**. Scale bar indicates 500 nm.

**FIGURE 5:**
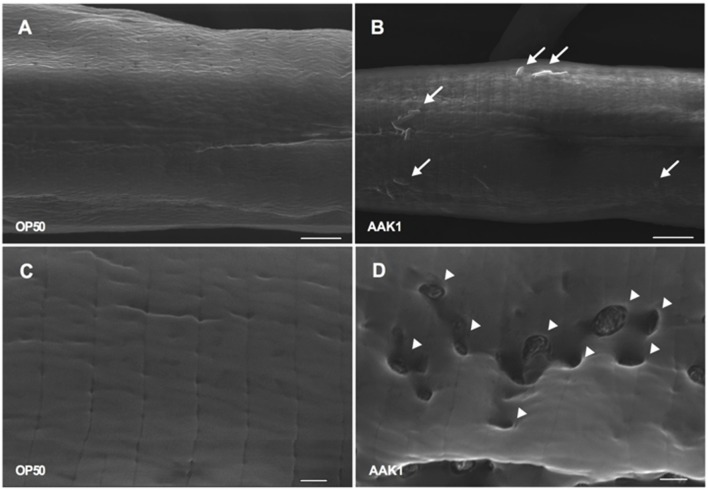
**Scanning electron microscopic analysis.** Features of N2 animals infected with *A. dhakensis* AAK1 (right panels, **B,D**) and control *E. coli* OP50 (left panels, **A,C**) for 48 h (upper panels) and 72 h (lower panels) observed by scanning electron microscopy. At 48 h, AAK1 cells that had adhered to animal surface are indicated by arrows **(B)**. Uneven surface and punctate lesions caused by invasion of AAK1 are indicated by arrowheads at 72 h **(D)**. Scale bars in the upper panel indicate 5 μm, and those in the lower panel indicate 1 μm.

The morphological changes in *C. elegans* with *A. dhakensis* AAK1 infection were observed by TEM (**Figure 4**) and SEM (**Figure 5**). Significant muscle damage appeared at 72 h. In TEM, wavy change, patch hypo-dense lesions, and loosening of myosin filaments were evident (**Figure 4D**) and SEM showed that the pathogens invaded *C. elegans* through the cuticle. At 48 h *A. dhakensis* AAK1 adhered to the worm surface (**Figure 5B**), on which obvious erosion and pole formation were seen at 72 h (**Figure 5D**). Taken together, our results show that *A. dhakensis* AAK1 can adhere to the surfaces of *C. elegans* and cause cuticle invasion as well as muscle damage.

### *Aeromonas dhakensis* Infection-Induced Muscle Necrosis and Rapid Death in *Caenorhabditis elegans*

**FIGURE 6:**
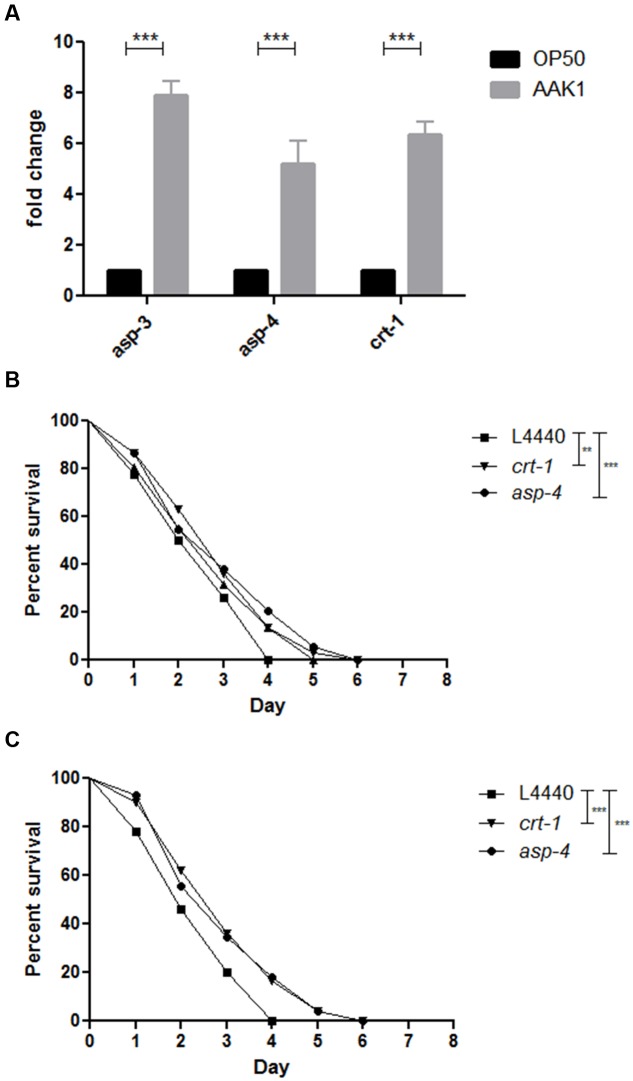
***Aeromonas dhakensis* induces necrosis in *C. elegans.*** **(A)** Relative expression levels of *asp-3*, *asp-4*, and *crt-1* genes measured by quantitative real-time PCR (qPCR) in *C. elegans* infected by *A. dhakensis* AAK1 for 72 h were higher than *C. elegans* fed by *E. coli* OP50. **(B)** The RNAi-sensitive worms, NL2099 *rrf-3 (pk1426*), with knockdown of *crt-1* or *asp-4* by RNAi showed significantly enhanced survival after *A. dhakensis* infection, relative to the control animals with RNAi control (L4440). **(C)** The WM118 worms with depressed expression of *crt-1* or *asp-4* by RNAi restricted to the muscle were more resistant to *A. dhakensis* infection than RNAi control (L4440). ^∗∗^*P* < 0.01; ^∗∗∗^*P* < 0.0001.

To validate whether *A. dhakensis* infection induces necrosis in *C. elegans*, we examined the expression of an array of necrosis genes, including *asp-3* and *asp-4* encoding aspartyl proteases, and *crt-1* encoding calreticulin (Syntichaki et al., 2002) separately, by q-PCR. At 72 h, relative expression levels of these genes in *C. elegans* infected by *A. dhakensis* AAK1 were higher than those by *E. coli* OP50 (**Figure 6A**). The increased expression of necrosis-associated genes with *A. dhakensis* infection was compatible to the necrosis phenotype observed in the morphological analysis. To confirm whether necrosis is a part of a bystander inducible mechanism or a deleterious consequence of infection, we studied the survival of *C. elegans* with depressed expression of *asp-4* or *crt-1* upon *A. dhakensis* infection. The RNAi sensitive worms, NL2099 *rrf-3 (pk1426*), with knockdown of *asp-4* or *crt-1* by RNAi showed significantly enhanced survival after *A. dhakensis* infection, relative to the control worms with RNAi empty vector L4440 (**Figure 6B**). The WM118 worms with suppressed expression of *asp-4* or *crt-1* by RNAi restricting to the muscles were also significantly resistant to *A. dhakensis* infection than control worms (*P* < 0.0001) (**Figure 6C**). The RNAi used in the experiment did not influence the growth of bacteria (**Supplementary Figure S1**). Taken together, we demonstrated that *A. dhakensis* infection induces muscle necrosis, which is associated with rapid death of infected worms.

### Virulence of Hemolysin-Deletion *A. dhakensis* to *C. elegans*

The virulence of a hemolysin-deletion mutant of AAK1 (AAK1 Δ*hlyA::Kan*^R^), which was produced by the methods described in the **Supplementary Materials**, was studied. Virulence of this mutant to *C. elegans* was significantly reduced when compared with the parent AAK1 strain in a survival analysis (**Supplementary Figure S2**). The severity of muscle damage induced by this mutant isolate in the RW1596 transgenic worms was less evident than that induced by AAK1 (**Supplementary Figure S3**). Similarly, the expressions of *crt-1*, *asp-4*, and *asp-3* induced by AAK1 in *C. elegans* at 72 h were significantly abolished in those treated by the AAK1 Δ*hlyA::Kan*^R^ mutant (all *P* < 0.0001). (**Supplementary Figure S4**).

## Discussion

Necrotizing muscle infection, a type of severe invasive infection, is usually associated with high morbidity and mortality in humans despite surgical intervention and antibiotic treatment (McHenry et al., 1995). The pathogenesis of severe muscle infection is not well understood. Here, we established a disease model of soft-tissue, namely muscle infection by *A. dhakensis* in *C. elegans*, and such a model can be used as a tool to study the pathogenesis of muscle necrosis. Traditionally, small mammal models, such as mice, rats, rabbits, or dogs, have provided valuable information to understand the pathophysiology of muscle necrosis caused by degenerative disease (Klyen et al., 2011), injury (Hargens et al., 1981; Daly et al., 2011), or infection (Hidalgo-Grass et al., 2004; Cavanaugh et al., 2009; Chatterjee et al., 2016). Among soft-tissue infection models, rats or mice were commonly adopted (Hidalgo-Grass et al., 2004; Cavanaugh et al., 2009; Chatterjee et al., 2016). However, the disadvantage of these infection models is that the results are not reproducible or sustainable due to the leakage and spread of bacteria from the inoculated tissues (Yoshioka et al., 2014). In addition, morphological analysis of the extent of muscle necrosis necessitates the sacrifice of large animals and is laborious work.

Over the past decade, increasing evidence has shown that *Drosophila*, zebrafish, and *C. elegans* are good models for studying soft-tissue pathology because they share conserved immune responses against bacterial or fungal infections in soft-tissues of mammals (Zugasti et al., 2014; Chatterjee et al., 2016). Out of these models, *C. elegans* is an acceptable model to explore innate defense strategies of epithelial tissues, as its epidermal cells possess cell-autonomous defense mechanisms against invading pathogens or physical injury (Ziegler et al., 2009; Zugasti et al., 2014; Zhang et al., 2015). From the temporal perspective, extensive tissue destruction usually evolves rapidly after bacterial invasion in the cases of necrotizing myonecrosis (Hakkarainen et al., 2014). Therefore, the innate immunity in muscle tissues should play an important role in the first-line host response, as it is activated after sensing pathogen-associated molecular patterns (Mogensen, 2009) or damage-associated molecular patterns at the early stage of infection (Chen and Nunez, 2010).

In the present study, *A. dhakensis* was chosen for testing in a disease model as it can cause a variety of forms of soft-tissue infection in humans. In some endemic areas, such as southern Taiwan, *A. dhakensis* is of clinical importance due to its virulence and antimicrobial resistance, and it is also the predominant species in wound infections (Chen et al., 2014a,b). In addition, the whole-genome sequence of the *A. dhakensis* strain AAK1 has been published (Wu et al., 2012), and genomic information of *C. elegans* is readily available, making further and systematic genetic studies of host-microbe interaction possible. Our study showed that *A. dhakensis* AAK1 can adhere to the surfaces of *C. elegans* and cause cuticle invasion as well as muscle damage. This phenomenon resembles necrotizing fasciitis or bacterial myositis in humans (Hiransuthikul et al., 2005; Kihiczak et al., 2006).

The pathogenicity of *Aeromonas* species has been considered to be multifactorial. A number of virulence factors, such as secretion systems, toxins, and the quorum sensing system (QSS), have been reported (Tomas, 2012; Chen et al., 2016). However, the pathogenesis of muscle necrosis caused by *A. dhakensis* infection remains unclear. Of the toxins discovered in *Aeromonas* species, hemolysin is one of the most important virulence factors and has been considered as a causative factor of human diarrheal disease (Burke et al., 1982). In a study of mouse infection model, intravenous injection of hemolysin can elicit lethality in mice (Asao et al., 1986). In addition, hemolysin is able to cause fluid accumulation in rabbit ligated ileal loop and infant mouse intestine *in vitro* (Asao et al., 1986), and hemolysin can produce inflammation of injected areas over the body surface of carps (Kanai and Takagi, 1986). In the present study, muscle necrosis caused by a hemolysin-depletion mutant of *A. dhakensis* was attenuated significantly when compared with parent strain. The results suggest hemolysin plays an important role in the pathogenicity of this species. Our study validate the potential of this model in studying the pathogenesis of muscle necrosis related to *Aeromonas* infections and provide a platform to find the potential therapeutic approaches to improve the outcome of severe *Aeromonas* soft-tissue infection.

Rapid death with necrosis of *C. elegans* is a characteristic of *A. dhakensis* infection. Mosser et al. (2015) found that *A. dhakensis* exhibited high virulence in the *C. elegans* infection model, with unusual presentation of rapid lysis of dead bodies by *A. dhakensis* BVH28b. A similar phenomenon was observed caused by our study strain, *A. dhakensis* AAK1 (Chen et al., 2014a). To our knowledge, these features are rarely reported in other pathogens. Other bacteria, such as *Enterococcus faecalis*, *Staphylococcus aureus*, *Pseudomonas aeruginosa*, *Salmonella typhimurium*, and *Serratia marcescens*, cause intestinal infections in *C. elegans* (Ermolaeva and Schumacher, 2014). For example, colonization of the host intestinal tract is a key virulent mechanism of *P. aeruginosa* in the “slow-killing” assay (Tan et al., 1999). In another example, Shiga-like toxin-producing enterohemorrhagic *E. coli* (EHEC) O157:H7 has been known to infect and kill *C. elegans* with concomitant demonstration of bacterial colonization and induction of characteristic attaching and effacing lesions in the intestinal epithelium of worms (Chou et al., 2013). In contrast to rapid necrosis induced by *A. dhakensis*, no significant morphological changes in *C. elegans* were observed after infection with EHEC O157:H7 at 72 h (**Supplementary Figure S5**). Likewise, the expression of necrosis-associated genes of *asp-3*, *asp-4*, and *crt-1* was not obviously changed with EHEC infection and was in accordance with morphological observation (**Supplementary Figure S6**). Instead, our SEM and TEM studies showed that *A. dhakensis* AAK1 attached to the worm skin and invaded at the initial infection site, at least during the time frame we analyzed. This evidence suggests the initiation of necrotic cell death is associated with recognition of the pathogen in the cuticle. The exact mechanism needs to be elucidated in the future.

Our data suggested that *A. dhakensis* infection causes necrotic cell death in muscle cells in *C. elegans*, and necrosis has a detrimental effect during *A. dhakensis* infection as necrosis-deficient worms were more resistant to bacterial infection than wild-type worms. Several mechanisms have been linked to necrotic cell death, such as autophagy, calcium, TOR-mediated nutrient sensing, and lysosomal proteases (Crook et al., 2013). Of the variety of necrotic mechanisms, homeostasis of calcium in the endoplasmic reticulum and calcium-dependent proteases may play important roles in necrotic death of muscle cells. It is well known that mitochondrial calcium overload is a general mechanism for muscle cell necrosis in various muscle diseases (Wrogemann and Pena, 1976). We discovered that host *asp* and *crt-1* genes encoding aspartyl proteases and calreticulin (a calcium-binding protein), respectively, implicated in cell necrosis, were upregulated by *A. dhakensis* infection. In addition, worms with muscle deficiency of *asp* and *crt-1* were more resistant to *A. dhakensis* infection. Xu et al. reported that calreticulin activation could induce calcium release from the endoplasmic reticulum (Xu et al., 2001) and downstream aspartyl proteases activity was associated with necrotic cell death and neurodegeneration in *C. elegans* (Syntichaki et al., 2002, 2005). Therefore, our data suggest that *A. dhakensis* infection may activate *crt-1* expression in muscles and promotes the release of calcium from the endoplasmic reticulum, activates the cascade of aspartyl proteases, and consequently causes necrotic cell death of worms.

To sum up, our work found that gross pathology and histopathology induced by *A. dhakensis* infection are similar in humans and *C. elegans*, thus validating the value of this model. The advantages of *C. elegans* were exploited to visualize gross and histological soft-tissue damage. In addition, the *C. elegans* model can be used to study host innate immunity and bacterial factors involved in soft-tissue infection. The application of the *C. elegans* model provides an opportunity for high-throughput genetic screening for pathogen or host factors involved in clinically important bacterial infections.

## Author Contributions

P-LC and Y-WC contributed equally to this work. P-LC, W-CK, and C-SC conceived and designed the experiments. C-CO and Y-WC performed the experiments. P-LC and C-SC analyzed the data. C-JW and T-ML contributed critical reagents and analysis tools. P-LC, W-CK, and C-SC wrote the paper.

## Conflict of Interest Statement

The authors declare that the research was conducted in the absence of any commercial or financial relationships that could be construed as a potential conflict of interest.

## Footnotes

1 http://wormbook.org/chapters/www_intromethodscellbiology/intromethodscellbiology.html\#d0e108

## Supplementary Material

**SUPPLEMENTARY MATERIALS |** An AAK1 strain with 1.8K base-pair hemolysin gene flanked by kanamycin resistance *nptII* gene was generated by marker exchange utilizing a homologous recombination with suicide plasmid carrying *sacB* gene. We utilized the conjugation method to deliver the construct plasmid into AAK1 with the aid of *E. coli* S17-1 λ πstrain. After 3 h of co-culture, the colonies grew on the plates of Aeromonas Selective Medium LabM 167 (Lab M; Lab M Ltd, Lancashire, UK) with kanamycin overnight cultured at 37°C were selected. Colony PCR was performed to check the presence of *sacB* gene in the AAK1 strain. At the same time, we transferred those colonies to grow on the LB plate with 6% sucrose and kanamycin at 20°C in order to let *sacB* convert sucrose to toxic metabolite, levans, and facilitate efficient chromosome exchange between the transconjugated plasmid and the chromosome. After 2–3 days of culture, all the candidates were confirmed by genomic DNA PCR using full-length hemolysin primers to confirm an hemolysin-deficient mutant, AAK1 *ΔhlyA::kan*^R^, was obtained.

**FIGURE S1 | Co-culture of *Escherichia coli* carrying different RNAi clones of *asp-3*, *asp-4*, and *crt-1* with *Aeromonas dhakensis* AAK1 for 24 h did not affect the growth of *A. dhakensis* AAK1 by measuring colony forming units (CFUs) on kanamycin selective medium.** L4440, an empty vector, was used as a negative control of RNAi.

**FIGURE S2 | A hemolysin-deficient isogenic mutant of AAK1, AAK1 Δ*hlyA::Kan*^R^, prolonged the survival curve of *Caenorhabditis elegans* (*P* < 0.0001).**

**FIGURE S3 | A hemolysin-deficient mutant of *A. dhakensis*, AAK1 Δ*hlyA::Kan*^R^did not cause muscle damage as parent strain.** Rupture of muscle fibers (arrowheads) was obvious in worms with *A. dhakensis* infection at 72 h (middle). In contrast, *E. coli* OP50 (left) and Δ*hlyA::Kan*^R^ AAK1 (right) did not cause muscle damage.

**FIGURE S4 | Relative expression levels of necrosis-associated genes in *C. elegans* infected by a hemolysin-deficient isogenic mutant of *A. dhakensis* AAK1.** In the mutant, expression levels of *crt-1*, *asp-3*, and *asp-4* at 72 h are significantly lower to those in wild-type AAK1 by quantitative real-time PCR (*^∗∗∗^P* < 0.0001).

**FIGURE S5 | Enterohemorrhagic *E. coli* O157:H7 did not induce rapid lysis of *C. elegans* as *A. dhakensis* did.**

**FIGURE S6 | Expression levels of necrosis genes, *asp-3*, *asp-4*, and *crt-1*, in *Caenorhabditis elegans* infected by enterohemorrhagic *E. coli* O157:H7 were significantly lower than those in *C. elegans* infected by *A. dhakensis*.** ^∗∗∗^*P* < 0.0001.
