# Supplementary figures and images for: A Disease Model of Muscle Necrosis Caused by Aeromonas dhakensis Infection in Caenorhabditis elegans
Source: Front Microbiol. 2017 Jan 4;7:2058. doi: 10.3389/fmicb.2016.02058 (PMC5209350; doi:10.3389/fmicb.2016.02058)

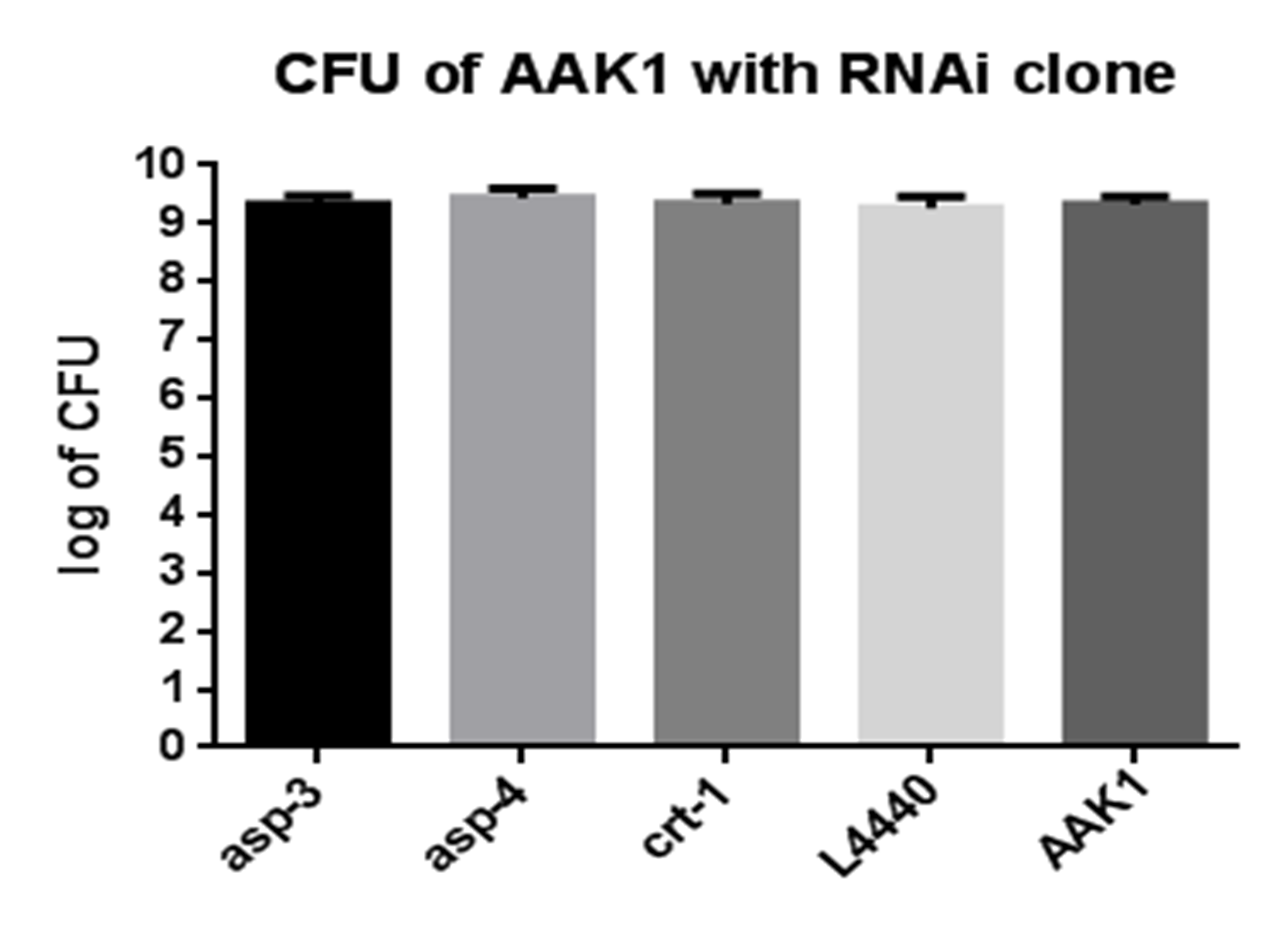

Supplement: Supplementary file 1 [file Image_1.TIF]

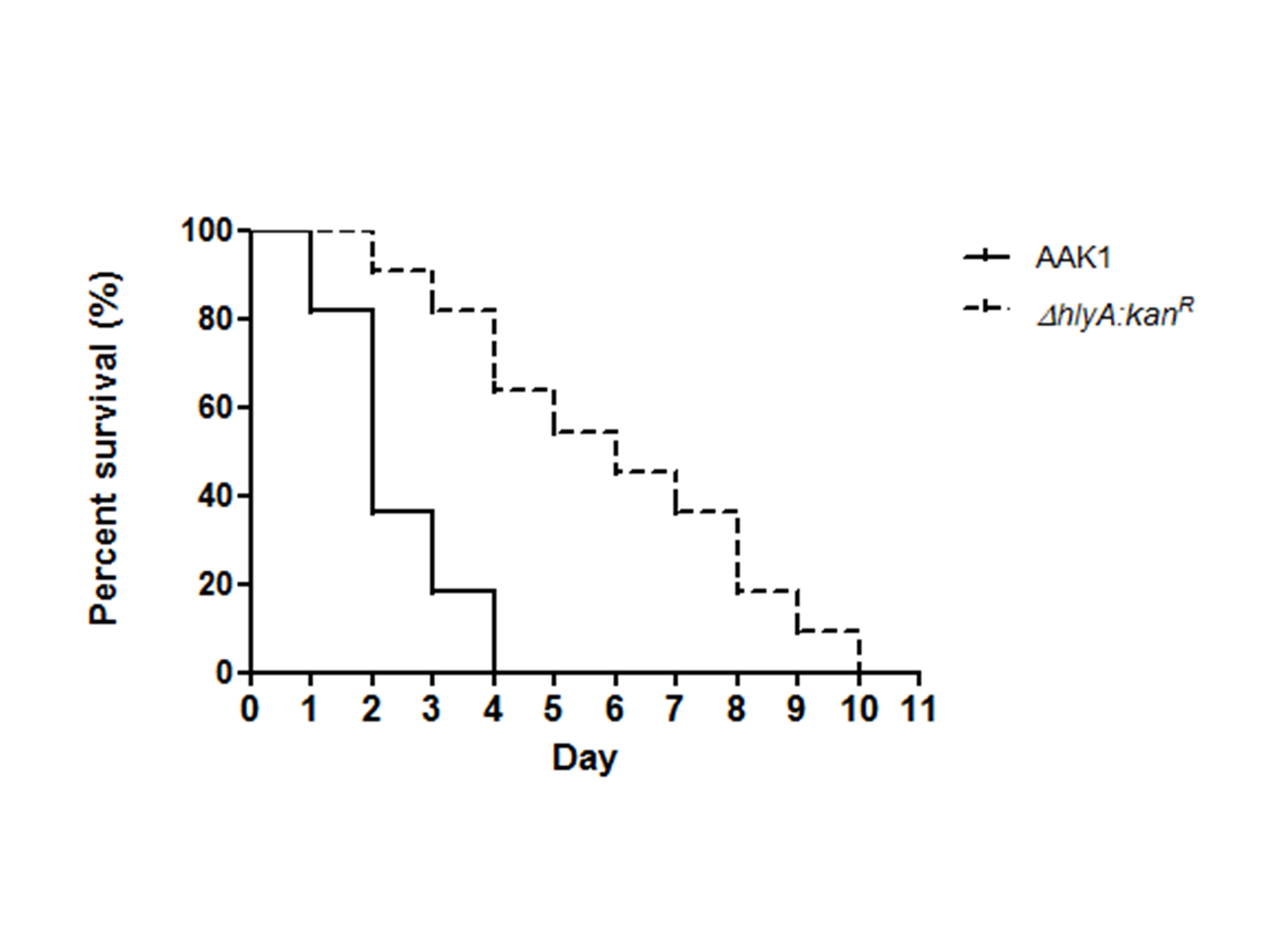

Supplement: Supplementary file 2 [file Image_2.TIF]

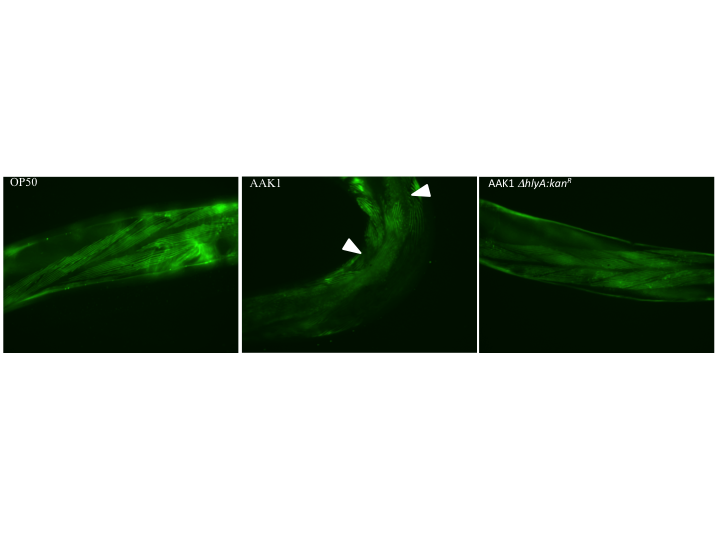

Supplement: Supplementary file 3 [file Image_3.TIFF]

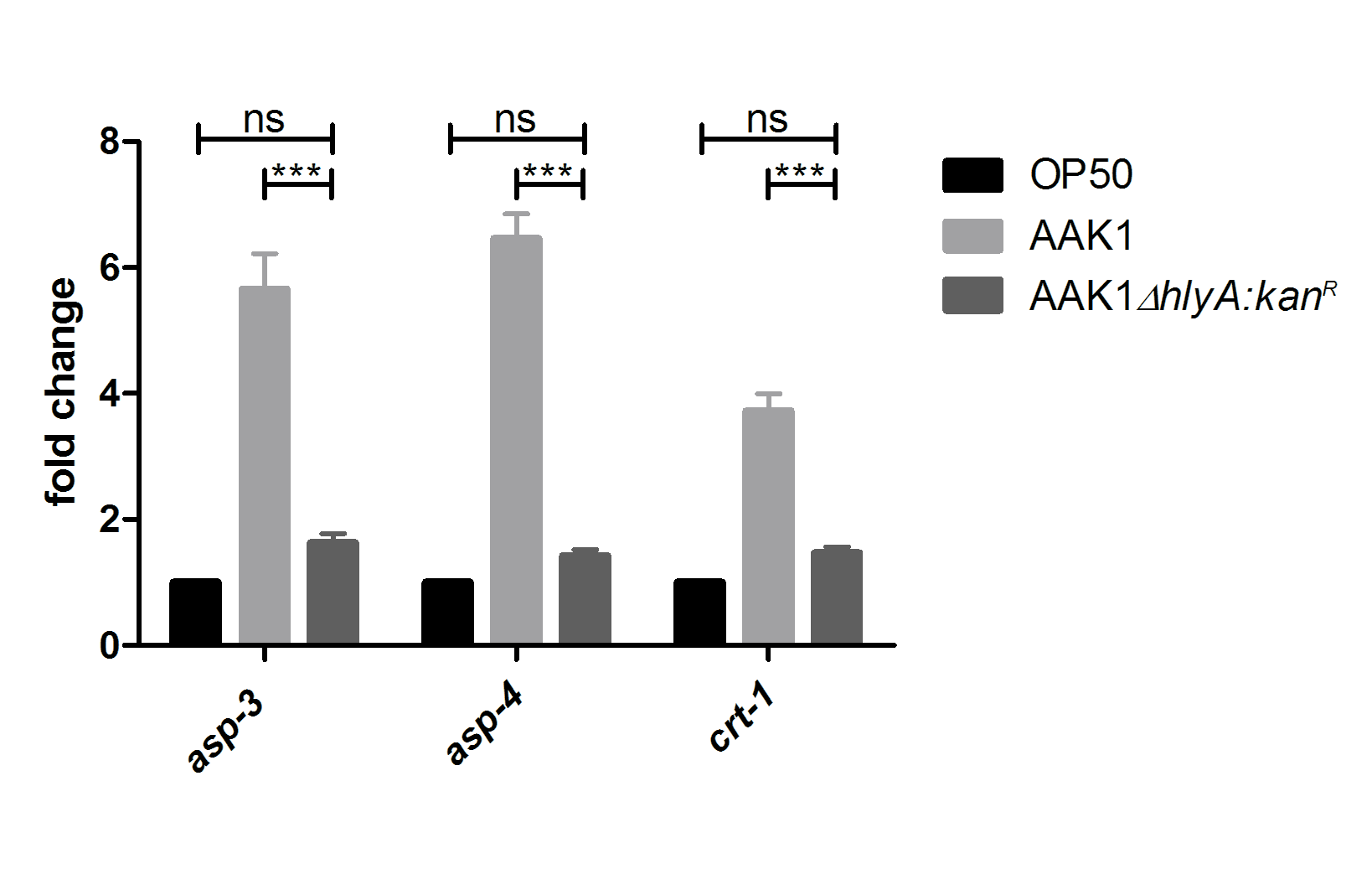

Supplement: Supplementary file 4 [file Image_4.tif]

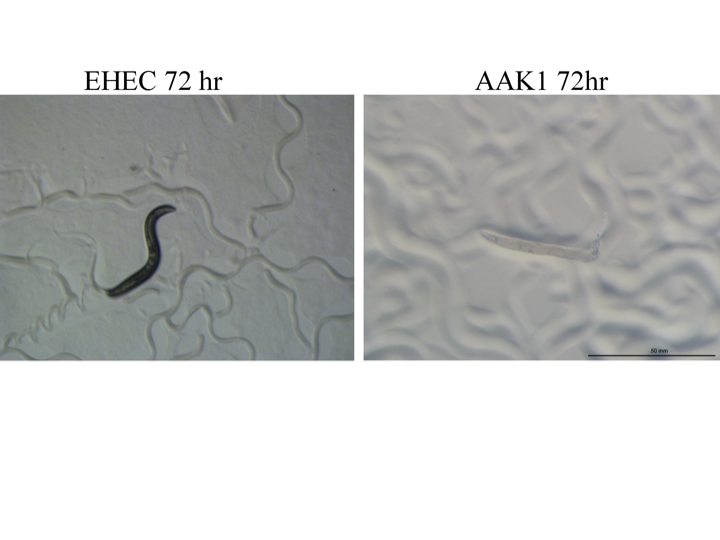

Supplement: Supplementary file 5 [file Image_5.TIFF]

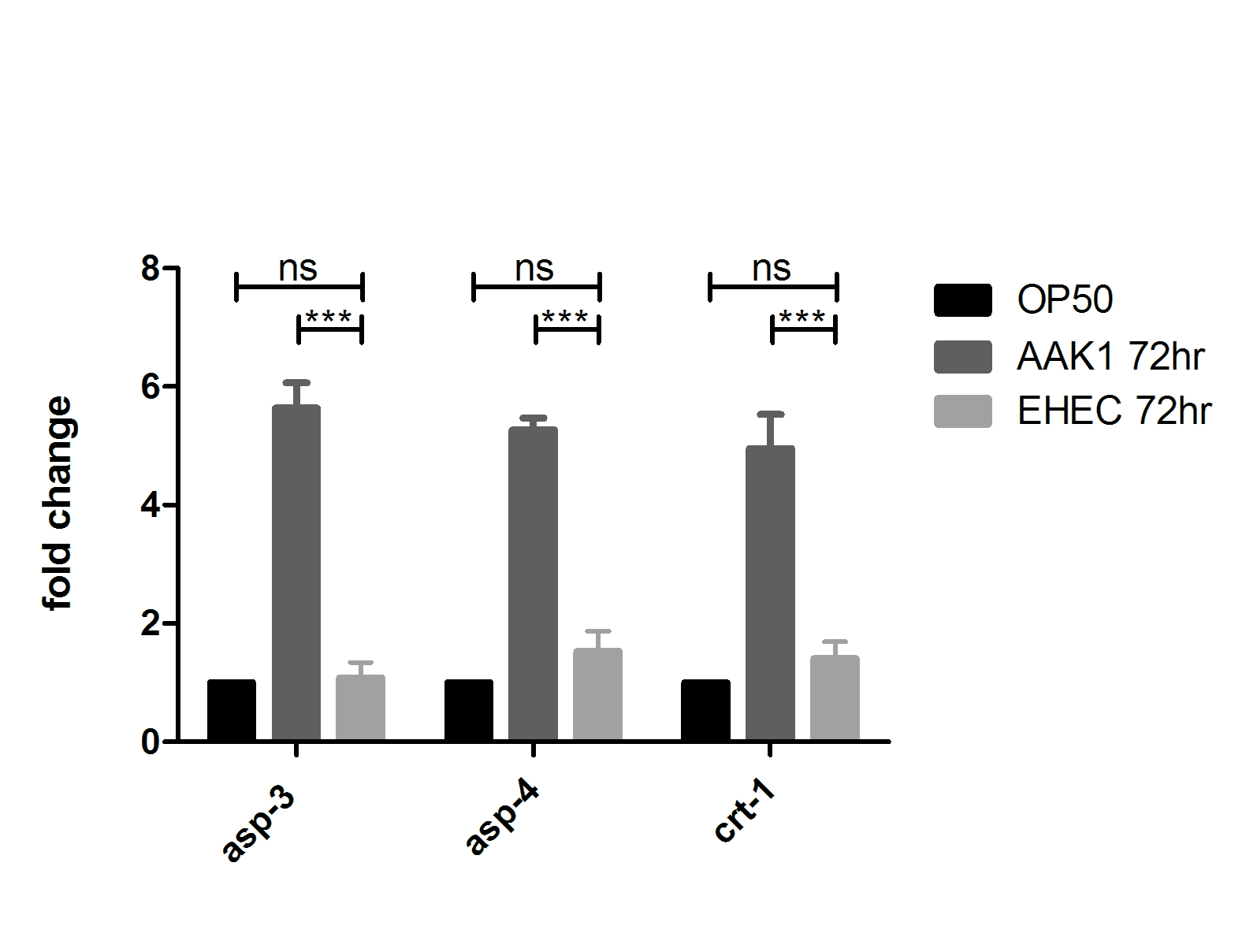

Supplement: Supplementary file 6 [file Image_6.tif]
